# Compressive sensing techniques based on secure data aggregation in WSNs

**DOI:** 10.1038/s41598-025-14959-0

**Published:** 2025-12-24

**Authors:** Marwa E. Madkour, Salah E. Soliman, Moawad I. Dessouky, Fathi E. Abd El-Samie, Mohammed E. Hammad, Amir S. Elsafrawey

**Affiliations:** 1 Nuclear Fuel Technology Department, Nuclear Fuel Management Division, Hot Labs Center, Egyptian Atomic Energy Authority, Cairo, Egypt; 2 Department of Electronics and Electrical Communications Engineering, Faculty of Electronic Engineering, Menoufia University, 32952 Menouf, Egypt; 3 Department of Information Technology, College of Computer and Information Sciences, Princess Nourah bint Abdulrahman University, P.O. Box 84428, 11671 Riyadh, Saudi Arabia; 4 Engineering Department, Nuclear Research Center, Egyptian Atomic Energy Authority, Cairo, Egypt

**Keywords:** Wireless sensor network, Security, Elliptic Curve Diffie-Hellman key exchange, PMLEACH routing, Elliptic curve cryptography, Engineering, Electrical and electronic engineering

## Abstract

This research paper presents an efficient data collection scheme for Wireless Sensor Networks (WSNs) that simultaneously compresses and encrypts sensor data to extend network lifespan. To address WSN resource limitations, the scheme combines Compressive Sensing (CS) with Elliptic Curve Cryptography (ECC) and Elliptic Curve Diffie–Hellman (ECDH) key exchange. Sensor data is securely compressed and encrypted using ECC-based public key mechanisms, mitigating CS-related attacks during aggregation and transmission. The measurement matrix seed serves as a private key that is exchangeed between sensor nodes and the base station, enhancing both security and efficiency. A prime-number-based Tree Path Identifier (TPID) routing and Cluster Head (CH) selection strategy is employed to optimize communication. Seven CS algorithms—including Orthogonal Matching Pursuit (OMP), Binary Compressive Sensing (BCS), Subspace Pursuits (SP), Approximate Message Passing (AMP), Split Bregman Iterative (SBI), Basis Pursuit (BP) and Compressive Sampling Matching Pursuit (CoSaMP) algorithms—are evaluated across various data sparsity levels. Results show that SP, AMP, and SBI algorithms outperform others in preserving energy, extending network life, and delaying the First Dead Node (FDN) appearance. Performance metrics include residual energy, network lifetime, total energy dissipation, and throughput. Energy savings confirm the superiority of the proposed hybrid scheme over traditional CS algorithms.

## Introduction

A data processing centre, often known as a Base Station (BS), and several dispersed sensors with limited resources make up a general WSN. In a single-hop topology, the sensor nodes send the gathered observation data straight to the BS via the wireless channel. In a multi-hop topology, data is transmitted through intermediary nodes known as the CHs, which are responsible for aggregating data from member nodes and forwarding it toward the BS. There are many WSNs based on CS applications, including imaging, video processing, cognitive radio networks, machine-type communications, and radar signal processing. In communication systems, physical-layer operations include channel estimation in wireless networks and channel estimation in power line communications^1^.

The WSN suffers from the energy-limited sensor nodes, which consume energy heavily depending upon the magnitude of data transmitted or received by the nodes in the network. In addition, there exist hotspot problems. Another significant issue with WSNs is that data is sent wirelessly over unprotected channels with low power and short transmission range, making it extremely easy for an adversary to intercept.

Two categories of encryption algorithms exist. One is based on private keys, while the other is based on public keys. Despite increased security provided by public-key encryption techniques such as ECC, Rivest–Shamir–Adleman (RSA), El-Gamal and Diffie–Hellman (DH), most public-key algorithms are not favored for usage on resource-constrained WSN devices^2,3. ^The ECC is an exception as it is adopted to provide large security with smaller key sizes. Private-key encryption techniques, such as Advanced Encryption Standard (AES), Data Encryption Standard (DES), Rivest Cipher 4 (RC4), Blowfish and Twofish, do not require a lot of computers or memory, but they demand that the keys are pre-stored on nodes that are vulnerable to attacks, when left in unguarded situations^4–6^.

We propose an effective security scheme aimed at ensuring safe and energy-efficient data collection in multi-hop-topology WSNs. In this approach, both the communicating parties (the sensor node and the BS) must share the CS matrix to enable CS-based compression. The sensor node uses the CS matrix to compress the data before transmission, and the BS applies it in a CS reconstruction algorithm to recover the original data. As is the case with all public-key algorithms, CS faces key sharing challenges, since exchanging the matrix over unsecure channel increases the vulnerability to security attacks. To address this issue, the ECDH key exchange technique is used for secure CS matrix seed sharing. By employing Pseudo-Random Number Generators (PRNGs) to produce the full CS matrix from the exchanged seed, the system ensures that the matrix remains unpredictable to attackers. Furthermore, the aggregated data received by CHs is encrypted using ECC. ECC is based on the algebraic structure of elliptic curves over finite fields. It provides high security with small key size, making it well-suited for resource-constrained WSN environments. It employs a public-private key pair, where the public key is used for encryption at the CH side and the private key is held by the BS for decryption. Although the public key is derived from the private key through elliptic curve point multiplication, it is computationally infeasible to deduce the private key from the public key. This combination of ECDH for secure CS measurement matrix seed exchange and ECC for encryption significantly enhances both the security and efficiency of the proposed scheme. 

Based on the integration of ECDH, ECC, and CS, the proposed scheme offers the following key contributions:

- Improving security by employing the ECDH technique to securely exchange the CS matrix seed between the BS and the WSN nodes once prior to transmission to determine the CS matrix.
- Avoiding direct CS measurement matrix exchange and avoiding also matrix transmission overhead, which enables the WSN nodes and the BS to generate their CS matrix independently, improving the security against CS-based security attacks.
- Integrating the ECC algorithm with CS during the transmission process between the CHs and the BS, strengthening reselience to CS attacks, while maintaining minimal energy consumption.
- Increasing security by addressing security risks to CS through the use of the ECDH key exchange technique and the key update mechanism, which allows the BS and WSN nodes to independently and dynamically regenerate the CS matrix in each transmission cycle.

For CH selection and cluster formation, the Prime-number-based Modified Low-Energy Adaptive Clustering Hierarchy (PMLEACH) algorithm is employed. The PMLEACH algorithm aims to distribute energy consumption in the WSN in a fair manner.

The remainder of this paper is structured as follows. Section II presents the literature review. The background knowledge is introduced in Section III. The proposed scheme is introduced in Section IV. Section V is devoted for experimental results. Section VI gives the conclusion.

## Literature review

ُECC algorithm and ECDH key exchange technique have been proposed for effective key management and distribution in WSNs^7^. Key pre-distribution techniques^8^ conserve energy and offer scalable storage capacity and efficiency. Approaches employing symmetric keys, such as the lightweight security scheme^9^ and the hierarchical clustered WSNs^10^, enhance security, scalability, and energy consumption. However, identical CS matrices generated by the BS and sensor nodes in each round may be vulnerable to known plain-text attacks^11^. Although these techniques have shown success in ensuring data privacy and security, their high computational complexity makes them less suitable for low-power computing, and limited-storage sensor nodes.

Another security technique^12^ evaluates CH nodes using a fitness parameter, incorporating direct, indirect, and historical trust influences. This trust calculation, however, increases computational requirements. The technique further selects a CH and a backup head using optimized coefficients. While utilizing multi-hop routing for data transmission from sensors to sink nodes, this technique lacks data compression at the CH. Additionally, the trust procedure and the Genetic Algorithms (GA) optimization introduce computational complexity. To mitigate these issues, compressing data from various sensors is crucial. The CS technique is well-suited for this task, combining sampling and compression in a single phase.

 Another security technique^13^ enhances intra-cluster and inter-cluster security. The intra-cluster security relies on fuzzy-logic-based trust evaluation by the CH, with secure nodes handling data transmission. Inter-cluster security depends on column transposition encryption, which maintains the data size. Our technique, however, employs a CS-based method for secure intra-cluster communication. It offers the advantage of achieving both compression and encryption, unlike the size-preserving encryption used in the inter-cluster phase.

The authors of^14^ proposed a multi-level, dynamic key management approach for homogeneous WSNs. This approach uses administrative keys to distribute communication keys, which in turn establishes secure communication channels between nodes. However, this hierarchical topology creates a hotspot problem near the BS. Nodes in this area become overloaded as all data passes through them, and the lack of compression exacerbates this issue.

In another work^15^, the network is initially divided into cells, with an agent node selected for each. Sensor node scheduling is based on the overlap of their sensing regions with neighbors. Cell agents calculate coverage and identify holes using this overlap data. Holes are then recovered using the grasshopper optimization technique and mobile nodes. However, due to various limiting factors, mobile nodes are often not a feasible option in most situations.

Within another framework^16^, the lightweight columnar transposition cipher technique is employed to guarantee the security of message exchange and to account for the processing limitations inherent in the nodes. The subsequent hop is chosen based on the parameters of node traffic, the distance to the BS, and the remaining energy. The routing procedure is executed in a hop-by-hop manner, with no compression being considered.

A significant effort has been exerted to accomplish data privacy, security, and energy efficiency by utilizing CS as a security scheme, irrespective of its security degree. A CS-based Security approach for Data Collection (SeDC) was proposed^17^, where the authors used CS across limited fields to lower the cost of data gathering and public-key encryption to address the issue of key distribution. The similarity between CS and lattices has been investigated. The network lifetime has been reduced, though; if additional calculations, such as encryption and compression, are performed at each node, they represent computationally demanding operations. Cipher-text attack and plain-text attack are analyzed under two different scenarios. Other authors^18 ^ used Even-Rodeh codes and ElGamal algorithm to compress and secure the data, respectively. The compression ratio and space savings are the variables investigated. Data security is provided via El-Gamal algorithm; however, the size of data from the El-Gamal encryption process is increased and the difficulty of solving the discrete algorithm problem is enlarged.

Other authors^19^ presented a routing protocol for load balancing and Quality of Service (QoS) enhancement based on GA optimization. This protocol accommodates sensor nodes with varying capabilities, such as energy levels, processing powers, or communication ranges. The GA optimization may require substantial computation for evaluating fitness functions and selecting CHs, which could be a burdensome for resource-constrained sensor nodes. The WSN faces security challenges based on CS encryption schemes. These challenges include brute-force attacks, fault injection attacks, side channel attacks and invalid curve attacks. To address these issues and achieve secure data collection, researchers have explored various approaches. CS-based systems combining compression and encryption have been used to lower data gathering costs and improve network performance^20^.

While much research has been proposed leveraging CS as a security scheme^21–25^, irrespective of its security level, to achieve data privacy, security, energy efficiency, and overall efficiency, our proposed work takes a different approach. In our scheme, node data is CS compressed and also inherently encrypted before being transmitted to the CH for aggregation and ECC encryption. Then, they are finally received by the BS.

## Background

### CS background

Increasing network lifetime depends on reducing overall energy consumption and ensuring a more balanced energy distribution among nodes. CS enhances energy efficiency, primarily through its inherent capability to reduce data volume through compression. It eliminates the need for traditional transforms in compression by mapping high-dimensional signals to lower-dimensional domains through projection onto an appropriate basis. The original signal can be reconstructed from compressed data. While transforms, such as Fourier transform, Discrete Cosine Transform (DCT), and wavelet transform, are not the main focus of this paper, they remain relevant for CS. Therefore, a brief overview of sparsity and signal representation is warranted.

When a compressible signal, such as temperature, humidity, carbon monoxide level, pollution level, air/water quality, forest fire or seismic data, is projected onto an appropriate basis, it decays fast, meaning that only a small portion of the transformed coefficients contain most of the signal energy. Many projection coefficients are zero or small enough to be neglected when the domain (basis) is properly set. A signal is ${\it{h}}$-sparse if it only contains ${\it{h}}$ non-zero coefficients. Moreover, a signal is compressible if a large number of projection coefficients are tiny enough to be neglected. If there are orthogonal bases provided by (${{\mathbf\uppsi}}_\mathrm{1},{{\mathbf\uppsi}}_\mathrm{2},{{\mathbf\uppsi}}_\mathrm{3},\dots\:\dots\:...{{\mathbf\uppsi}}_{\it{N}}$), we can express $\mathbf{x}$ as a $\it{k}$-sparse signal in $\mathbf{\uppsi}$ as in Eq. (1). In the following equations, the vectors are denoted by boldface lowercase letters, while boldface capital letters represent matrices.

1 $$\:\mathbf{x}\:=\:\mathbf{\uppsi\:}\mathbf{k}$$

If $\mathbf{x}\in\:{\mathbb{R}}^{\mathit{N}}$ describes the sparse signal to be compressed, in the CS framework, compression of $\mathbf{x}$ is performed using the following linear operation:

2 $$\:{\left[\mathbf{y}\right]}_{\mathit{M}\times\:1}\:=\:{\left[\boldsymbol{\Phi\:}\right]}_{\mathit{M}\times\:\mathit{N}}{\left[\mathbf{x}\right]}_{\mathit{N}\times\:1}$$

$\left[\mathbf{y}\right]$ : The compressed signal.

$\left[\mathbf{x}\right]$ : The signal vector to be compressed.

$\left[\mathbf{\Phi}\right]$ : The projection or measurement matrix.

The CS concept means that a sparse or compressible signal $\mathbf{x}$ of dimension $\it{N}$ with ($\it{M}\ll\:\it{N}$) can be transformed into $\mathbf{y}$ utilizing $\it{M}$ linear projections as in the Eq. (3).

3 $$\:\mathbf{y}\:=\:\mathbf{\Phi\:}\mathbf{x}=\mathbf{\Phi\:}\mathbf{\Psi\:}\mathbf{k}=\mathbf{\Theta\:}\mathbf{k}$$

$\text{where }\:\mathbf{\Phi\:}\mathbf{\Psi\:}=\mathbf{\Theta}$. It is possible to reconstruct $\mathbf{x}$ under certain conditions on the measurement matrix $\mathbf{\Phi}$. If $\mathbf{x}$ is sufficiently sparse, the first direct solution is obtained by solving the following optimization problem.

4 $$\:\mathit{m}\mathit{i}\mathit{n}{\parallel\:\widehat{\mathbf{x}}\parallel\:}_{0}\:\:\mathit{s}.\mathit{t}.\:\mathbf{y}=\:\mathbf{\Phi\:}\widehat{\mathbf{x}}$$

Equation (4) describes a non-convex combinatorial optimization problem. This problem needs extensive searches over subsets of the measurement matrix columns. For example, if a sparse signal has $\mathit{h}$ none-zero values and has a total dimension equal to *N*, then we have ${\mathit{h}}^{\mathit{N}}$ subsets that exceed the normal capability of calculation. Linear programming is used by convex algorithms to handle the convex optimization problem to recover the compressed signal, as it is computationally more efficient. Several approaches have been proposed like greedy, iterative thresholding, combinatorial/sublinear, non-convex optimization, Bayesian, and Bregman iterative algorithms^26^.

### Elliptic curve cryptography background

In cryptography, a key is a sequence of characters utilized in encryption algorithms to transform data in such a way that it appears random^27^. ECC is commonly applied in three areas: Elliptic Curve Digital Signature Algorithm (ECDSA), encryption (ECC) and key exchange (ECDH).

- ECC uses algebraic curves over finite field ${\mathbb{F}}_\mathit{p}$ to generate keys to be used by the parties. Also, both parties need to agree on an elliptic curve beforehand. In mathematics, an elliptic curve can be described as a plane algebraic curve as given by an equation of the form ${\mathbb{E}}_\mathit{p}(\mathit{a},\mathit{b})$ :

$$\:\mathit{y}^{2}=\:\mathit{x}^{3}+\:\mathit{a}\mathit{x}\:+\:\mathit{b}\:\:\:\mathrm{m}\mathrm{o}\mathrm{d}\left(\mathit{p}\right)$$

**Fig. 1:**
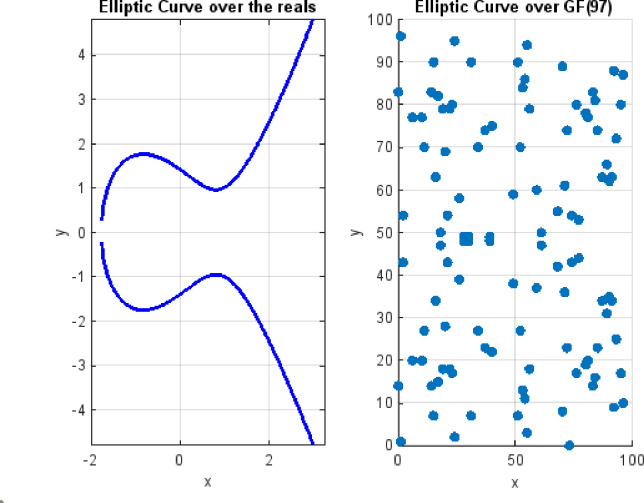
(a) Elliptic curve over the real numbers. (b) Elliptic curve over the Galois field GF(p), modulo $\mathit{p}$.

$\mathrm{w}\mathrm{h}\mathrm{e}\mathrm{r}\mathrm{e}\:\:4\mathit{a}^{3}\:+\:27\mathit{b}^{2}\ne\:\:0$. This ensures that the graph is non-singular, and thus a tangent line can be identified at every point, which is important for point duplication. Figure 1a illustrates the elliptic curve ${\mathbb{E}}_\mathit{p}(-\text{2, 2})$ over the real numbers, showing its smooth, continuous shape. Figure 1b shows the same curve defined over the Galois Field GF(*p* = 97), where all coordinates are integers between 0 and − 1, and arithmetic operations are performed *modulo* $\mathit{p}$. On the other hand, curves over real numbers are continuous and require floating-point calculations. The discrete versions of curves over GF(*p*) avoid rounding and precision issues, making them ideal for digital computations and cryptographic applications.

The ECC offers a comparable level of cryptography to that obtained with RSA, but with noticeable reduced key size. The Discrete Logarithm Problem (DLP) for an elliptic curve is defined as for two points ${\mathit{Q}}_{1}$ and ${\mathit{Q}}_{2}$ on eliptic curve surface to find a positive integer $\mathit{n}$, such that ${\mathit{Q}}_{2}=\mathit{n}\times{\mathit{Q}}_{1}$, and $\mathit{n}$ represents the private key. It is important to note that certain elliptic curves are considered more secure than others, and therefore the selection should be made with caution and based on established security criteria.

The ECDH key exchange is a key agreement technique that enables two parties, each possessing elliptic curve parameters, to establish a shared secret key over an insecure channel. It is an asymmetric key exchange protocol based on ECC to enable the sensor node and the BS to securely establish a shared secret key over an insecure channel, which can then be used for CS compression. In ECDH, each party generates its own private key, and the private key must remain confidential. The ECDH operates by multiplying one party private key with the other party public key to compute a shared key. This shared key is then used for CS matrix generation. To illustrate how this works, this scenario can be explained as follows:

Two parties, Ax and Bx, first agree on the elliptic curve domain parameters, including the curve equation over a finite field ${\mathbb{E}}_{\mathit{p}}(\mathit{a},\mathit{b})$, a prime number* p*, and a base point *G*; $\mathit{G}\in{\mathbb{E}}_\mathit{p}(\mathit{a},\mathit{b})$ will be used in the subsequent key exchange process.

- Ax selects a secret value $\mathit\alpha$, computes $\mathit{A}=\mathit\alpha\mathit\:G$, and sends $\mathit{A}$ to Bx.
- Bx also selects a secret value $\mathit\beta$, calculates $\mathit{B}=\mathit\beta\mathit\:G$, and sends $\mathit{B}$ to Ax.
- Ax then calculates the $Public\:key=\mathit\alpha\mathit{B}$ to determine the Diffie-Hellman key, and Bx does the same and gets $\mathit\beta\mathit{A}$.

Ax key = $\mathit\alpha\mathit{B}$ = $\mathit\alpha$ $(\mathit\beta\mathit{G})$ = $\mathit\beta\left(\mathit\alpha\mathit{G}\right)$ = $\mathit\beta\mathit{A}$ = Bx key. Both Ax and Bx end up with the same key. All the above calculations are implemented in $mod\left(\mathit{p}\right)$.

The ECC algorithm is used at each CH to encrypt the received data before transmission to the BS. First, we compute the public key ${\mathit{E}}_{pu}={\it{E}}_{\mathit{p}\mathit{r}}\mathit{G}$. Second, the message encryption is performed using ${\it{E}}_{\it{p}\it{u}}$. Let *W* be the message aggregated by the CH. This message needs to be represented on the curve. Then, we select *q* randomly from $0<\mathit{q}<\mathit{p}-1$. So, the encrypted message is;

5 $$\:\mathit{Z}=[\mathit{c}_{1},\mathit{c}_{2}]=[\mathit{q}\times\mathit{G},\mathit{W}+(\mathit{q}\times{\mathit{E}}_\mathit{pu}\left)\right]$$

where *c*_1_ and *c*_2_ are the encrypted messages. The message decryption is performed at the BS using the private key.

6 $$\:\mathit{W}=[\mathit{c}_{2}-{\mathit{E}}_{\mathit{p}\mathit{r}}\mathit{c}_{1}]$$

### WSN structure

**Fig. 2:**
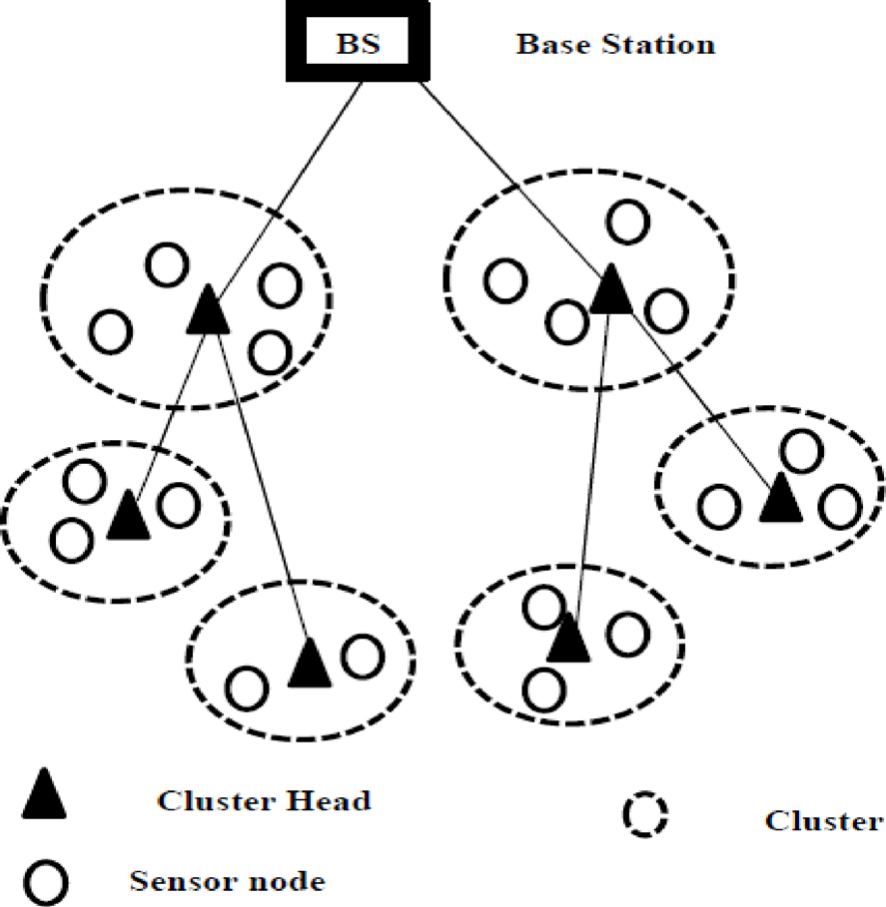
WSN structure.

A WSN is made up of spatially-dispersed sensors and one or more BS. In our scenario, only one BS exists. Sensors monitor physical factors such as temperature, vibration, or motion in real time and generate sensory data. A sensor node can act as both a data source and a data router. A BS, on the other hand, gathers information from sensors. In environmental monitoring applications, sensors may be required to continuously communicate data to the BS to provide real-time monitoring of the environment. This mode ensures that the BS always has the latest measurements from the sensor nodes. It is suitable for critical real-time monitoring applications, such as temperature control and industrial process monitoring. In event-driven mode, communication is triggered by the detection of anomalies or predefined conditions. The WSN deployment can be of mesh, star, or tree type. Tree structure has many advantages like solving the hotspot problem. Figures 2 shows the tree WSN architecture.

Wireless data transmission through insecure channels makes it relatively simple for an attacker to listen to the conversation. As a result, the primary issues for WSNs are security and energy conservation. Security can be improved by encrypting the data sent between sensor nodes and the BS. The CS compression and reconstruction processes can be performed in parallel with encryption and decryption. As a result, data compression and encryption are carried out concurrently, which increases energy efficiency.

### Tree path identifier (TPID)

The TPID plays a crucial role in ensuring efficient routing within network topologies, especially those structured as spanning trees or hierarchical trees. Each node in the network is assigned a prime-numbered address, and the paths in the routing tree are identified using TPIDs. A TPID is obtained by multiplying the node addresses along the path, resulting in a unique ID for each path based on the prime addresses of the nodes. By decomposing the TPID, each node along the path can be identified, and each node can determine its own path based on its TPID. This approach ensures that each path is uniquely identified and facilitates efficient routing within the network. Its significance can be summarized as follows:

1. Unique Path Identification: TPID provides a unique identifier for each path within the tree, allowing routers or switches to quickly recognize and differentiate between different routes. This uniqueness helps prevent routing ambiguities and loops.
2. Efficient Route Computation: By encoding the path information, TPID enables rapid determination of the shortest or most optimal route without extensive computation or lookups. Devices can use TPID to quickly match incoming packets to their corresponding paths.
3. Simplified Routing Tables: Incorporating TPID reduces the size and complexity of routing tables. Instead of storing extensive path details, routers can store TPIDs, which serve as compact representations of paths, leading to shorter lookup times.
4. Loop Prevention and Path Validation: TPID assists in detecting and preventing routing loops by verifying whether a path has been previously traversed or is valid. This improves network stability and reliability.
5. Fast Convergence: During topology changes like link failures, TPIDs enable quick recalculation and dissemination of new paths, reducing convergence time and maintaining network performance.
6. Support of Hierarchical Routing and Scalability: In large networks, TPID helps manage hierarchical routing schemes efficiently by encapsulating path information, making the network scalable and easier to manage.

The TPID is vital for ensuring that routing decisions are made efficiently, accurately, and reliably by providing a compact, unique, and recognizable representation of paths within a tree-structured network. This enhances the overall network performance, scalability, and robustness.

## System model

### Two challenging scenarios

- If each node generates two keys, namely the public key and the private key, the node can share its public key with others for communication purposes, while the private key is used for decryption. However, this process is computationally intensive and requires a significant amount of energy. Therefore, it is not a suitable solution to be applied on low-power WSN devices.
- If the BS generates both the public and private keys and transmits the public key to the entire network, each node can use it to encrypt its data. However, when it comes to data aggregation at the CH, the CH needs to decrypt the data, perform aggregation, and encrypt it again for transmission to the BS. This process results in increased energy consumption for the CHs and requires the CHs to possess the private key. This poses security concerns as the use of a public-key algorithm in this manner becomes insecure.

### Challenge solutions

- The integration of the CS scheme, which combines encryption and compression, with ECC as a public key algorithm enables CS to address the aggregation issue without requiring a private key at the CH.
- An ECDH key exchange technique is introduced to address the CS matrix key distribution problem (i.e., distributing the measurement matrix seed), enabling secure and efficient exchange of pseudo-random keys between the BS and the sensor nodes.
- A novel approach is introduced to strengthen the security of the CS farmwork, providing resilience against potential security attacks.

### The proposed scheme stages

The proposed scheme consists of three main stages: Setup, Secure data gathering and Data reconstruction. In the Setup stage, efficient clustering and routing trees are established by using the properties of prime numbers to minimize power consumption during data transmission. The Secure data gathering stage depends on a CS-based approach to perform simultaneous data compression and encryption with ECC at CHs. Finally, the Data reconstruction stage depends on an efficient algorithm that accurately recovers the original sensor data from the compressed measurements, thereby improving the data reconstruction performance.

1. Setup

The proposed scheme leverages the principles of prime number theory to construct a routing tree that supports multi-hop routing from CHs to the BS, thereby improving the power efficiency of WSNs.

**Base station network routing process**

In the BS, a routing tree is constructed and **TPID**s are assigned to each node. The sequence of operations is as follows:

1. **Initial Setup**:

- Each node starts by assigning itself a large number as its level and setting its parent as null.
- The BS assigns itself a level of 0 and a **TPID** of 1.
- The BS then broadcasts a **BUILD** message to its immediate neighbours. This message contains the BS **ID**, **level**, **TPID**, and **energy level**.

2. **Upon Receiving a BUILD Message**: When a node receives a **BUILD** message, it performs the following actions:

- If the node current level is greater than the level in the received **BUILD** message:

- It adds the sender address to its candidate parent set **C**.
- It updates its level to be one more than the received level (**BUILD**.level + 1).
- The node then calculates its TPID as the product of its own address and the sender TPID. The sender becomes the node parent, selected based on having the highest energy level.

(b) If the node level is less than or equal to the received **BUILD** level:

- It ignores the message.

(c) After updating, the node broadcasts a **BUILD** message containing its **ID**, **level**, **TPID**, and **energy level** to its neighbors, but it only does this once.

3. **Subsequent Updates**: In each subsequent round, the node updates its **TPID** by selecting a parent from its candidate set **C**, specifically the parent with the highest remaining energy. If multiple candidates have the same energy level, the node chooses the one with lower **TPID**.

**Packet forwarding process**

To forward a packet from a source node to a destination node, the system calculates the difference between the TPID of the source (s: **TPID**) and the **TPID** of the destination (d: **TPID**):

- **If the difference is positive** (d: **TPID** - s: **TPID** > 0):
  - The packet is forwarded down to a child node.
  - If mod(d: **TPID**, c:**TPID**) = 0 (i.e., the destination **TPID** is divisible by the child **TPID**), the packet continues toward its destination.
  - If mod(d: **TPID**, c:**TPID**) ≠ 0 or the node has no children, the packet is discarded.
- **If the difference is negative** (d: **TPID** - s: **TPID** < 0):
  - The packet is forwarded to the parent node of the current node.
- **If the difference is zero** (d: **TPID** - s: **TPID** = 0):
  - The packet has reached its destination, and it belongs to node d.

This process enables dynamic tree-based routing in the network, using TPIDs and energy-based parent selection for efficient packet forwarding.

The PMLEACH is used for CH selection and cluster creation. It is an improved algorithm with the aim of achieving fair distribution of expended energy in the WSN. This is based on the unique prime factorization theorem, which states that any natural number can be represented as the product of powers of prime numbers^28^. The CH election and cluster formation process of PMLEACH is as explained below:

2) CH Election and Cluster Formation

Each sensor node $\mathit{s}$ selects a random number $\mathit{t}_\mathit{r}$; $0\le\:\mathit{s}.\mathit{t}_\mathit{r}\le\:1$. This value is compared with a predefined threshold value *T*. Nodes with random numbers below *T* become cluster members, while those with values above *T *are selected as CH nodes. In the first round, all node energies are equal. In subsequent rounds, an energy model is applied taking into account the distance to the BS $\mathit{d_\mathit{to\:BS}}$ and the residual energy parameters. Selecting CH nodes based on both their distances to BS and their remaining energies is crucial for optimizing energy consumption and prolonging network lifetime. A cost function is implemented to manage these factors, effectively.

$$\mathit{s}.\mathit{CostFunction}=\mathit\alpha\:\times\:\mathit{s}.\mathit{E}_\mathit{remaining\:energy}+\mathit\beta\:\times\:(\mathit{s}.\mathit{d}_\mathit{to\:BS})^{-1}$$

To determine the values of $\mathit\alpha\; \mathrm{and} \;\:\mathit\beta$, the variation of energy is considered at each round. This variation is quantified using the standard deviation of the residual energy. Finally, the threshold value *T* for a node $\mathit{s}$ is calculated as:

$$\mathit{s.T}\: = \left\{ {\begin{array}{*{20}c} {\mathit{p} \times \:(\mathit{s}.\mathit{CostFunction})^{2} \times \:(1 - \mathit{P }\times \:\mathit{mod}(\mathit{r},\left\lfloor \mathit{p^{{ - 1}} } \right\rfloor ))^{{ - 1}} \:} & {\:if\:\:\mathit\psi\ \left( \mathit{s,r} \right) = 0} \\ {0\;\;\;\;\;\;\;\;\;\;\;\;\;\;\;\;\;\;\;\;\;\;\;\;\;\;\;\;\;\;\;\;\;\;\;\;\;\;\;\;\;\;\;\;\;\;\;\;\;\;\;\;\;\;\;\;\;\;\;\;\;\;\;\;} & {if\:\:\mathit\psi \:\left( \mathit{s,r} \right) = 1} \\ \end{array} } \right.$$

where $\mathit{p}$ represents the desired percentage of CHs, and $\mathit\psi$ denotes the eligibility function that determines whether a node will participate in the CH selection process during the current round *r*. $\mathit\psi\left(\mathit{s},\mathit{r}\right)=0$ means that node *s* is eligible to act as a CH at round *r*; if $\mathit\psi\left(\mathit{s},\mathit{r}\right)=1$, node *s* is not eligible in that round. To determine weather a node is eligible to participate in the CH selection process in the current round, each node selects a prime number *q* (*q* > 2); and then calculates the eligibility function $\mathit\psi\left(\mathit{s},\mathit{r}\right)$ as follows:

$$\:\mathit\psi\left(\mathit{s},\mathit{r}\right)=\left\{\begin{array}{c}1,\:\:\:if\:\mathit{mod}(\mathit{r},\:\mathit{q})\:=\:0\:\mathrm{or}\:0\:\le\:\:\mathit{mod}(\mathit{r},\:\mathit{q}^{2})\le\:\:\mathit{q}\:+\:1\\\:0,\:\:\:\:\:\:\:\:\:\:\:\:\:\:\:\:\:\:\:\:\:\:\:\:\:\:\:\:\:\:\:\:\:\:\:\:\:\:\:\:\:\:\:\:\:\:\:\:\:\:\:\:\:\:\:\:\:\:\:\:\:\:\:\:\:\:\:\:\:\:\:\:\:\:\:\mathrm{otherwise}\:\end{array}\right.\:$$

Cluster formation proceeds as follows. Each normal (non-CH) node *s* joins the CH ($\mathit{CH}_\mathit{i}$) that satisfies the two conditions: (1) The distance between node *s* and $\mathit{CH}_\mathit{i}$ is less than the distance between node *s* and BS, (2) The distance between $\mathit{CH}_{i}$ and BS is less than the distance between node *s *and BS. If no selected CH satisfies both conditions for the node *s,* the node joins the nearest CH among the selected CHs.

3) Secure Data Gathering

The proposed security framework operates in two integrated levels. At the node level, CS-based encryption is applied at each non-CH node using the ECDH key-sharing method, which securely exchanges the sensing matrix $\mathbf{\Phi}$ seed between the BS and the non-CH node. The security of this level lies in the fact that the sensing matrix, containing pseudo-random values derived from the exchange of CS keys (seed) remains inaccessible to attackers.

This CS-based encryptionprocess achieves two main objectives; minimizing the volume of transmitted data, and protecting the transmitted data against potential adversaries.

At the cluster level, each CH uses the BS public key to encrypt the data received from its member nodes using ECC before transmitting to the BS.

The primary goal of this stage is to ensure the confidentiality and integrity of all sensor data transmitted between the sensor node and the BS. To achive this target, ECC is combined with the ECDH public-key exchange technique, completing a secure and efficient end-to-end communication process.

At the BS, two types of keys are generated: (i) the CS-matrix seed, established in the first round using ECDH key exchange with each node, and (ii) the ECC private and public keys for the CHs. For the CS key, the seed is generated based on a Gaussian distribution sequence. PRNG requires an initial value, or seed, denoted as$\:{\:\mathit{g}}_\mathit{0}$, which is generated at the BS and securely transmitted to each node using the ECDH technique.Using the same seed at both the sensor node and the BS ensures the generation of an identical measuring matrix **Φ** for encryption and decryption. However, if an adversary was able to guess the seed, it can reconstruct the same matrix and compromise security. To prevent this, the proposed scheme employs a robust seed generation strategy that makes guessing computationally infeasible. In subsequent rounds, the seed generation at both sensor node and the BS is updated using the following equation. 

7 $$\:\mathit{g}_{n+1}=\:\mathit{b}_\mathit{d}\times\:\mathit{g}_{n}\times\left(1-{g}_{n}\right)\:\:\:\:\:;{b}_{d}\ne\:0$$

In subsequent rounds, the BS generates a new ECC key pair ${\mathit{E}}_\mathit{pu},\:{\mathit{E}}_\mathit{pr}$, and securely sends the corresponding public key to each CH.

*The algorithm steps*

**Node Side**:

- Each node $\mathit{i}$ receives the seed value $\mathit{g}_{0i}$ using the ECDH key exchange technique.
- Each node $\mathit{i}$ uses the seed value to generate its ${\mathbf{\varPhi}}_{\mathit{i}}$ matrix and perform compression operation ${\mathbf{y}}_\mathit{i}={\mathbf{x}}_\mathit{i}{\mathbf{\Phi}}_{\mathit{i}}$ by Eq. (3).
- Each node $\mathit{i}$ uses the seed to generate the secret value $\mathit{f}_\mathit{i}={\mathit{g}_{0\mathit{i}}}^{-1}$.
- Each node $\mathit{i}$ calculates the secure ${\stackrel{\prime }{\mathbf{y}}}_\mathit{i}=\mathit{f}_\mathit{i} \times{\mathbf{y}}_\mathit{i}$, and then sends ${\stackrel{\prime }{\mathbf{y}}}_\mathit{i}$ to its $\mathit{CH}_\mathit{i}$.

**CH Side**:

- Each $\mathit{CH}_\mathit{i}$ aggregates the data from its member nodes as $\mathbf{W}_\mathit{i}=\stackrel{\prime }{{\mathbf{y}}_{1}}+\stackrel{\prime }{{\mathbf{y}}_{2}}+\stackrel{\prime }{{\mathbf{y}}_{3}}+\dots\:\dots\:\stackrel{\prime }{{\mathbf{y}}_\mathit{l}}$, where $\mathit{l}$ is the number of nodes in the cluster$\:\mathit{i}$.
- Each *CH,* uses the public key ${\mathit{E}}_\mathit{pui}$ to encrypt the aggregated data $\mathbf{W}_\mathit{i}$, producing the ciphertext $\mathbf{Z}_\mathit{i}$ according to equation (5) .

**BS Side**:

- The BS uses its private key ${\mathit{E}}_\mathit{pri}$ to decrypt the received ciphertext $\mathbf{Z}_\mathit{i}$ from each $\mathit{CH}_\mathit{i}$, obtaining the aggregated data $\mathbf{W}_\mathit{i}$ according to equation (6). .
- BS calculates the secret key *f*_i_ and recomputes **y** from **y'** ${\mathbf{y}}_\mathit{i}={\stackrel{\prime }{\mathbf{y}}}_\mathit{i}/\mathit{f}_\mathit{i}\:$.
- The BS uses the generated seed $\mathit{g}_{0\mathit{i}}$ and generates the corresponding CS matrix ${\mathbf{\varPhi}}_{\mathit{i}}$.
- Finally, the BS recovers the original data by applying the agreed reconstruction algorithm by Eq. (4).

4) Data Reconstruction

Seven algorithms are employed for testing sparse signal recovery efficiency, representing different categories of recovery. BP algorithm represents a convex relaxation, while CoSaMP, SP, and OMP represent greedy algorithms. AMP is selected to represent the iterative thresholding category. The Bayesian approach is exemplified through the Relevance Vector Machine (RVM). SBI is used to represent the Bregman iterative algorithms. Heavy hitters on steroids and Iterative Re-weighted Least Squares (IRLS) are excluded from the simulation results due to their prolonged reconstruction times, rendering them unsuitable for real-time WSN applications.

## Performance evaluation

A WSN network with 100 sensor nodes is simulated to assess the performance of the proposed algorithms. The nodes are randomly deployed within a $(200\:m\times200\:m)$ area, with their locations recorded to ensure that all algorithms are evaluated under identical spatial configurations. The BS is located at the geometric center of the network. For performance evaluation, the network parameters are defined as follows:

- The nodes are static, battery-powered, and initialized with 2J of energy per node.
- Each node is assigned a unique prime-numbered address^28^.
- Date transmissions occurs under ideal channel conditions, and is assumed to be collision-free; the simulation runs for 2000 rounds.
- Each node acquires a sparse signal of length = 512, containing values in the range [0, spikes of 1], and transmits the compressed data to its corresponding CH.

PMLEACH routing algorithm^28^ is employed for organizing the network nodes into clusters, with each cluster comprising one CH and multiple Cluster Members (CMs). During each simulation round, each CM transmits its sensed compressed data to its respective CH¸ which aggregates and encrypts the received data; and forwards the aggregated and encrypted data to the BS. OMP algorithm that was utilized for compressive sensing in^29^ is also applied to the cluster network, and its performance is compared with those of the proposed algorithms. The evaluation considers multiple sparsity levels, and the compression performance metric is used to compare the behavior of the OMP with those of other algorithms. The compression ratio and the compression performance are calculated as:

- Compression Ratio = Output Data Size/Input Data Size.
- Compression Performance (%) = 100 × (1-Compression Ratio). It represents the percentage reduction in data size achieved through compression.

**Fig. 3:**
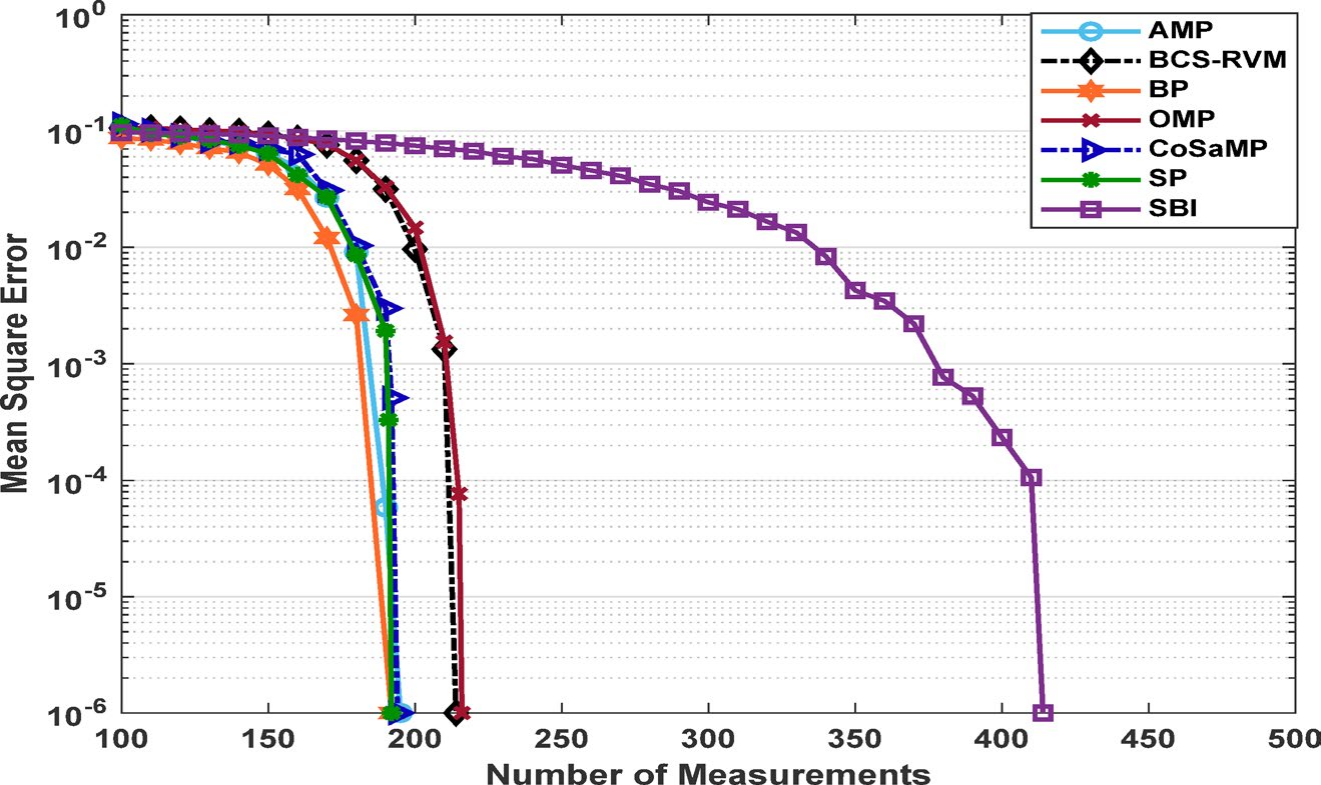
MSE for sparsity level 50 for various CS-based algorithms.

**Fig. 4:**
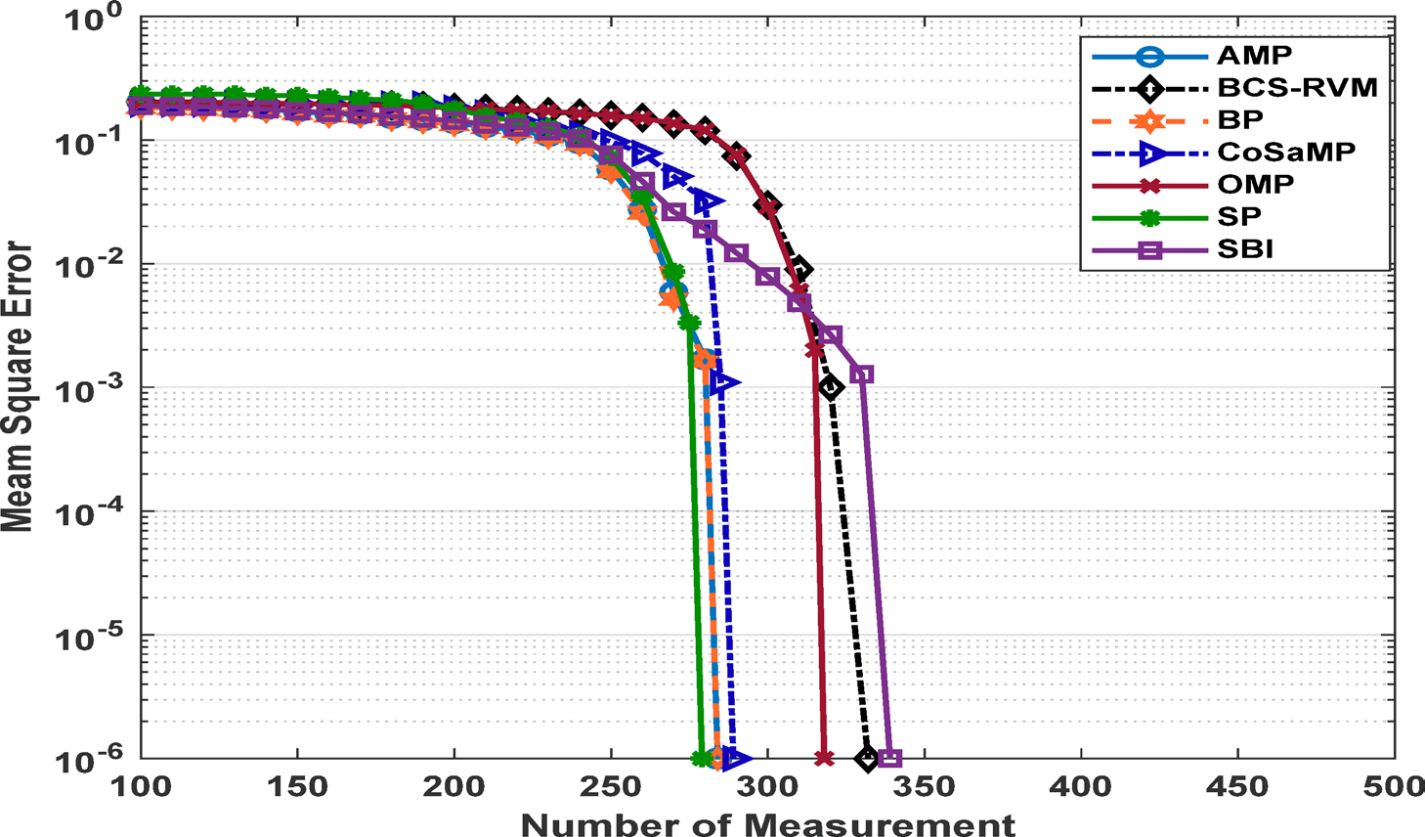
MSE for sparsity level 100 for various CS-based algorithms.

Figures 3 and 4 present the estimated Mean Square Error (MSE) of the reconstructed data for different compression ratios of the applied CS-based algorithms under two sparsity levels. For a sparsity level of 50, the SBI algorithm exhibits the poorest performance, with reconstruction error increasing significantly when the compression performance exceeds 20%. The BP algorithm demonstrates good performance, achieving the lowest MSE at a compression performance of 62.5%. The SP, AMP and CoSaMP algorithms also achieve results close to those of the BP algorithm. The OMP algorithm achieves an acceptable MSE up to a compression performance of only 57.8%.

For a sparsity level of 100, a slight improvement is observed in the performance of the SBI algorithm, which achieves an acceptable MSE with a compression ratio up to 33.7%. In contrast, the behavior of the OMP and BCS-RVM algorithms declines, operating effectively only at compression performance levels up to 37.9%, and 35.2%, respectively. AMP and BP algorithms achieve the best results for this sparsity level, sustaining an acceptable MSE up to 44.5%. The SP and CoSaMP algorithms achieve comparable compression performance levels of 45.5%, and 43.6%, respectively. The SP and CoSaMP algorithms, as shown in Figs 3 and 4, achieve approximately similar behavior for both low and moderate sparsity levels as they belong to the same category of greedy algorithms and their nearly identical algorithmic steps, differing mainly in their execution orders and pruning strategies.

**Fig. 5:**
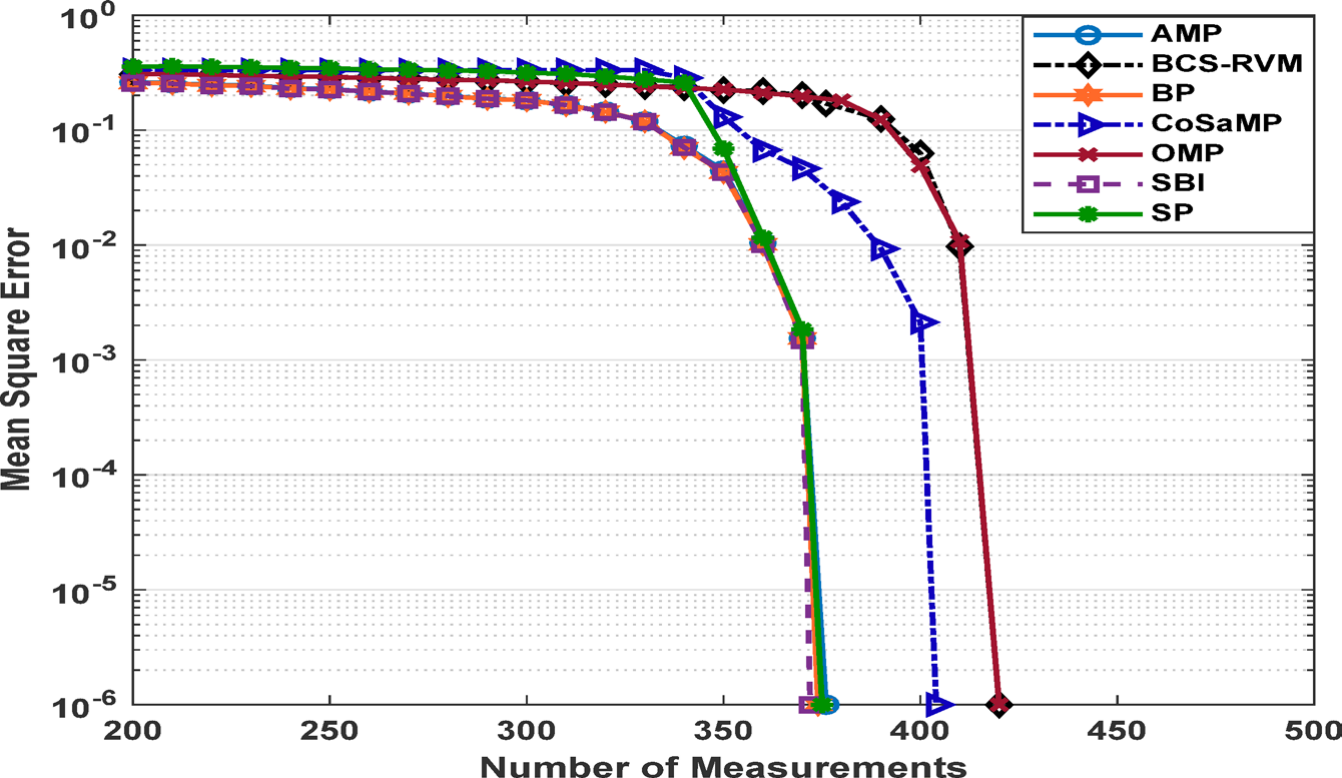
MSE for sparsity level 170 for various CS-based algorithms.

**Fig. 6:**
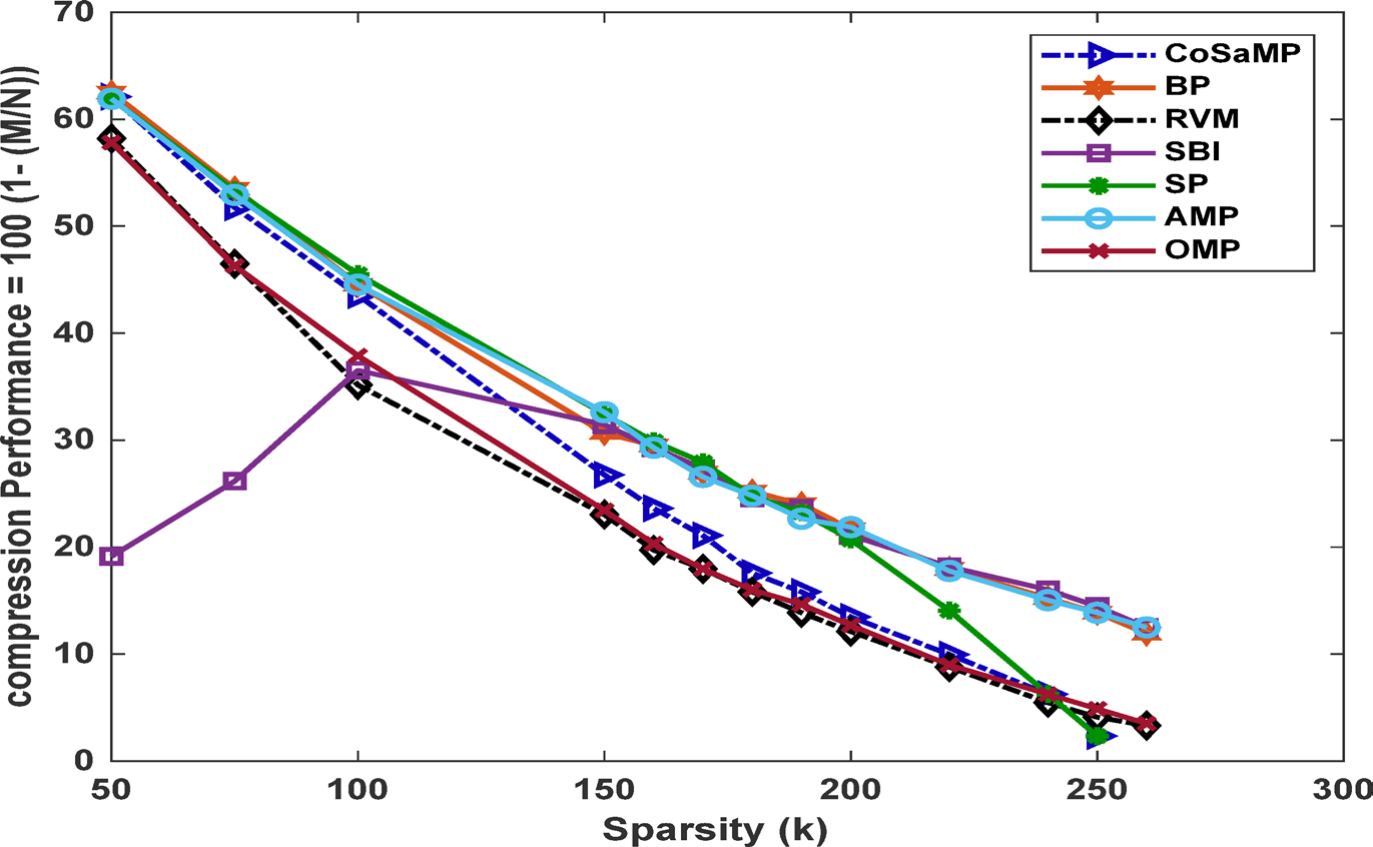
Compression performance versus sparsity for various CS-based algorithms.

Another scenario is presented in Fig. 5. For a sparsity level of 170, the response of the OMP algorithm closely follows that of the BCS-RVM algorithm with respect to variations in the compression ratio. Both algorithms exhibit the poorest performance, achieving an acceptable MSE value only for a compression performance up to 18%. In contrast, the SP, SBI, BP, and AMP algorithms achieve an improved performance, sustaining acceptable MSE levels for compression performance levels of 26.7%, 27.3%, 26.95%, and 26.56%, respectively. The CoSaMP algorithm achieves a compression performance of 21.1%, placing its performance level between the two groups.

Figure 6 illustrates the optimum compression performance for each applied algorithm that yields an acceptable MSE for different sparsity levels. The optimum compression ratio is defined as the maximum ratio that satisfies the minimum required MSE threshold, which in this study is set to $\:\:1\times\:{e}^{-6}$. This threshold ensures correct recovery of the received signal, though it may vary depending on the application. As shown in Fig. 6, an inverse relationship exists between the sparsity level and the achievable compression performance, as the sparsity value increases, the number of measurements required also increase, thereby reducing the attainable compression performance.

**Fig. 7:**
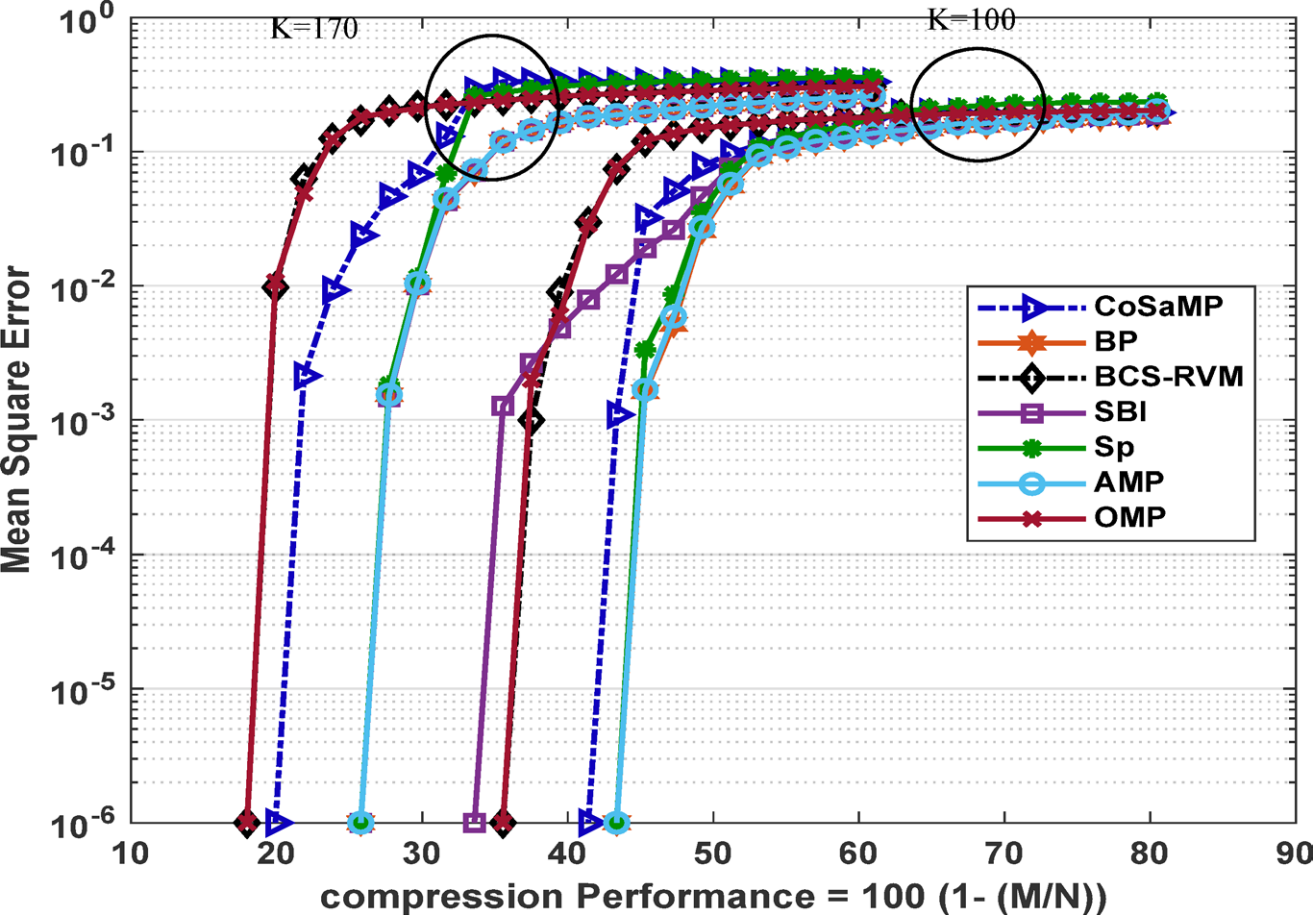
MSE versus compression performance for various CS-based algorithms.

The relation between MSE and compression performance for various CS-based algorithms is presented in Fig. 7. As the sparsity level increases, the compression performance decreases, since a larger number of measurements is required to meet the specified MSE threshold.

The following analysis reveals the effect of the compression achieved by the different algorithms on the key network metrics, namely residual energy, network lifetime, total energy dissipated, and network throughput. The analysis assumes uniform time intervals for data transmission, where each time unit on the time axis corresponds to a single transmission round. The compressed data length for each applied algorithm is obtained from Fig. 5 for a sparsity level of 170. It is evaluated at an MSE threshold of $1\times\:{e}^{-6}$.

**Fig. 8:**
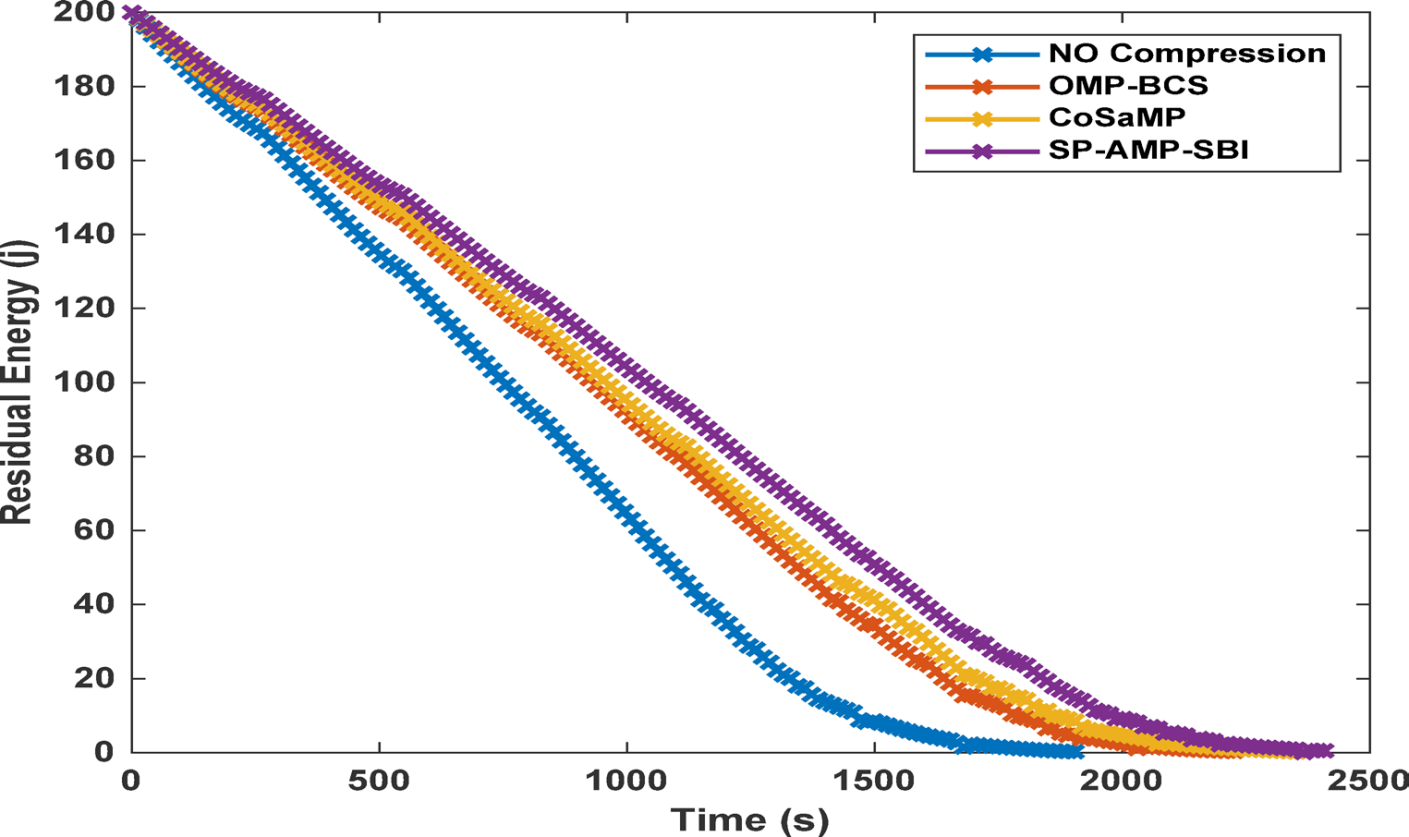
Network residual energy per round for different CS-based algorithms at a sparsity of 170.

**Fig. 9:**
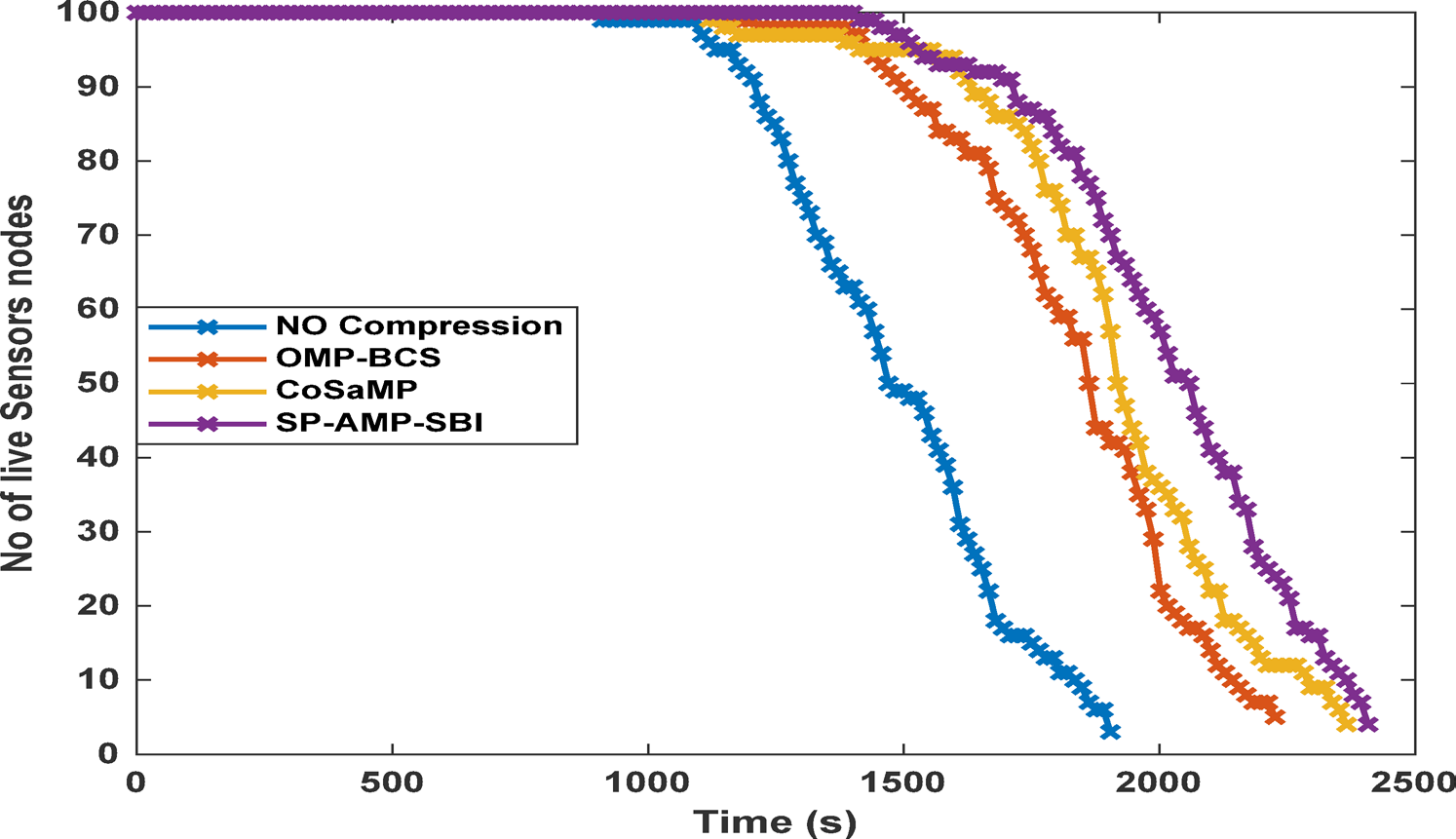
Network lifetime for different CS-based algorithms at a sparsity of 170.

**Fig. 10:**
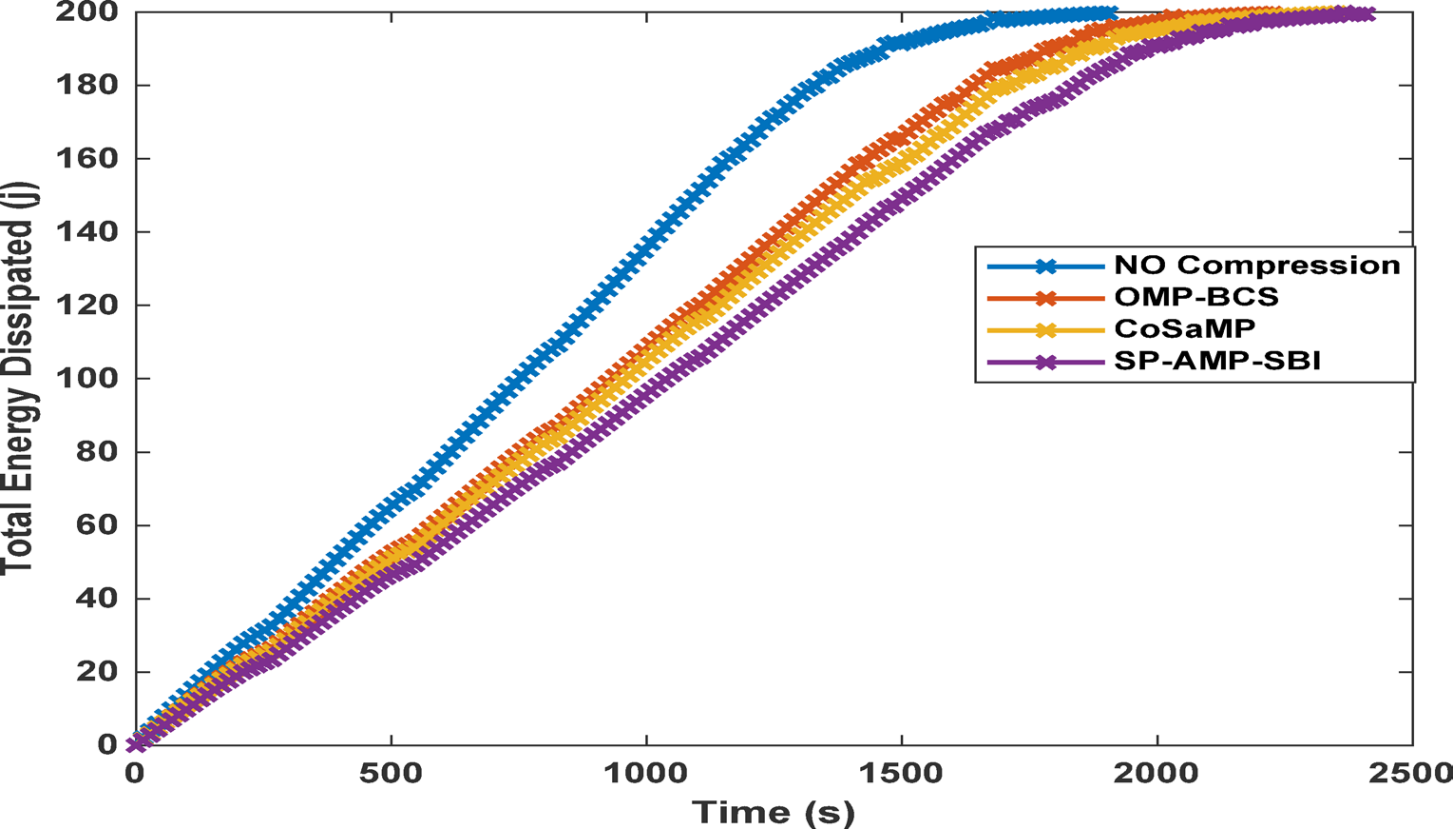
Total energy dissipated per round for different CS-based algorithms at a sparsity of 170.

**Fig. 11:**
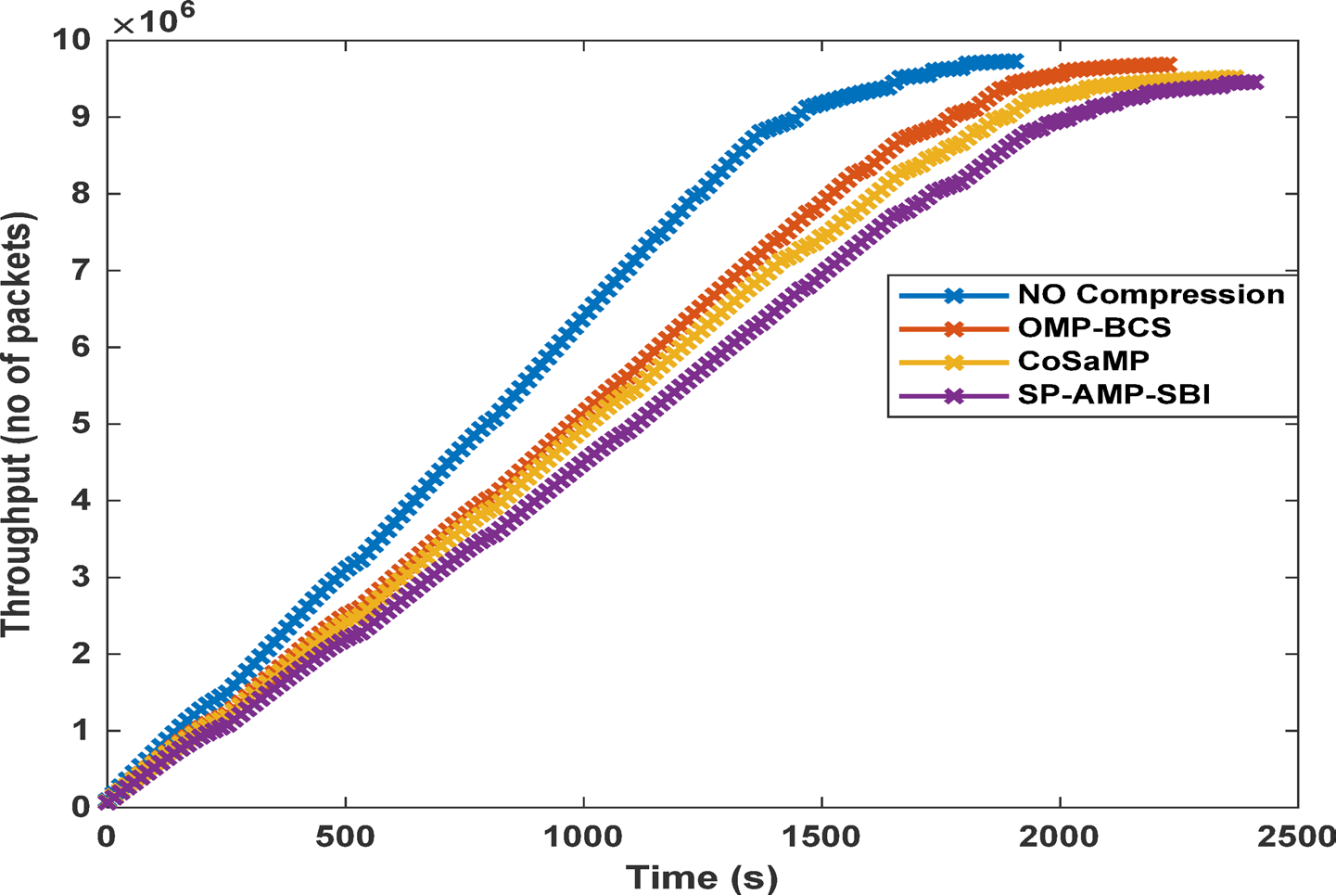
Network throughput per round for different CS-based algorithms at a sparsity of 170.

The first observed impact of the achieved compression is on the network residual energy per round, as illustrated in Fig. 8. The results clearly indicate that transmitting sensor data without compression leads to rapid depletion of network energy. In contrast, applying traditional CS with OMP and BCS algorithms improve the energy preservation. Further enhancement in the residual energy is observed, when employing the proposed CS algorithms, particularly the SP, AMP, and SBI algorithms, which dissipate less energy per round of network operation.

The next observed impact is on the network life time and FDN , as illustrated in Fig. 9. This parameter reflects the duration for which the network can operate with all nodes active without losing one of them. The energy savings achieved through data compression are directly reflected in the extended network life time. The no-CS case exhibits the shortest lifetime followed by the OMP and BCS CS-based cases. In contrast, the SP, AMP, and SBI algorithms not only prolong the overall network life time, but also delay the occurrence of the first node failure to significantly later rounds. The CoSaMP algorithm yields a moderate network life time.

Energy efficiency results are presented in Fig. 10, where the total energy dissipated in the no-CS case is the highest, followed by those of the OMP and BCS cases. The SP, AMP, and SBI algorithms achieve the lowest energy dissipation, while the CoSaMP algorithm performs moderately.

Finally, a measure of the network throughput, representing the total amount of successfully transmitted data, is also illustrated in Fig. 11. The CS-based SP, AMP, and SBI algorithms are capable of delivering the same network information, while significantly reducing the data size compared to both OMP and BCS algorithms and the case of transmitting uncompressed data.

## Conclusion

WSNs face critical challenges in security, data routing, and handling large-scale data. Leveraging the capability of CS to perform simultaneous sampling and compression, the associated measurement matrix – when kept confidential – can additionally act as a cryptographic element, thereby providing an inherent layer of data encryption. The proposed approach combines data compression, encryption, and a public-key sharing algorithm to achieve high levels of security and energy efficiency in addition to a minimal communication cost. This is achieved through the integration of encryption and compression of sensor data using a CS-based scheme, and the ECDH key-sharing technique. Additionally, the proposed scheme depends on ECC that is a public-key algorithm for encrypting CH data without requiring the private key, thereby mitigating attacks during the data transmission.. As a result, the proposed scheme outperforms other CS systems in terms of security and operational life span of cluster-based WSNs. The performance efficiency of the proposed scheme is evaluated through simulation analysis. Simulation results demonstrate that the proposed scheme reduces energy consumption, and extends network lifetime. The ECC depends on DLP, however any cryptographic system may be vulnerable to various types of attacks. Brute-force attack is intended to find the private key by trying all possible combinations. ECC solves this problem by using large key sizes and standardized curves. In side-channel attack, the attacker finds the private key by monitoring the side channel leaked information like power consumption, timing, and electromagnetic radiation during the execution of the encryption algorithm. This problem is solved by using a secure hardware. In fault injection attack, the attacker tries to introduce faults in the ECC calculation process. If successful, the attacker reveals a part of the private key. ECC mitigates fault injection attack and invalid curve attack by using a trusted elliptic curve and well tested algorithms and libraries that are resistant to various types of attacks. On the other hand, if a single private key is used for multiple transmissions, the attacker can exploit the poor key management practices to gain unauthorized access. Moreover, ECC is considered secure against classical computers, but it may become vulnerable to quantum computers in the future. Hence, ECC algorithm need to be updated according to the latest updates in quantum computing. Finally, in the implementation flow, errors in the implementation of the ECC algorithm can be exploited by the attacker, as in the case of poor random generator, insufficient entropy or programming errors. So, a proper and robust random generator is necessary. Future work will examine the impact of the number of keys on the system performance and the security level.

## Data Availability

The data that supports the findings of this paper are available on request from the corresponding author.
